# Laparoscopic port site Richter's hernia after robot‐assisted radical prostatectomy

**DOI:** 10.1002/iju5.12526

**Published:** 2022-08-23

**Authors:** Taisuke Tobe, Keiji Yuen, Tomihiko Yasufuku, Riku Uematsu, Akira Miyazaki, Masuo Yamashita

**Affiliations:** ^1^ Department of Urology Shiko Hospital Kobe Japan; ^2^ Department of Urology Hyogo cancer center Akashi Japan

**Keywords:** incisional hernia, postoperative complications, prostate cancer, robotic surgical procedures

## Abstract

**Introduction:**

Postoperative small bowel obstruction is a rare complication. One of its less frequent causes is port site hernia. We report a case of Richter's port site hernia in a patient who underwent robot‐assisted radical prostatectomy.

**Case presentation:**

A 73‐year‐old man who underwent robot‐assisted radical prostatectomy noted acute abdominal pain and nausea on the 11th postoperative day. Computed tomography scans revealed dilated small bowel loops. Adhesive ileus was initially suspected, which was relieved with conservative management, including ileus tube insertion. However, his symptoms worsened. Thus, a laparotomy was performed. The camera port wound was reopened, and the repaired fascia and small intestine were found incarcerated into the peritoneal defects. These findings were consistent with Richter's hernia.

**Conclusion:**

Port site hernia was not detected on computed tomography scans. Patients presenting with small bowel obstruction following laparoscopic surgery should be evaluated for port site hernia, and surgical management should be considered.

**Keynote message**

Port site hernia is one of the causes of postoperative bowel obstruction. An accurate diagnosis is key to early surgical intervention. In particular, Richter's hernias are difficult to diagnose. In cases where the area of strangulation is not apparent, conservative treatment may be given; however, surgery should still be considered.

**Abbreviations & Acronyms**
CT: computed tomography
RARP: robot‐assisted radical prostatectomy
PSH: port site hernia

## Introduction

RARP is the standard surgical procedure for localized prostate cancer. Postoperative small bowel obstruction is a rare surgical complication primarily caused by adhesions.

PSH is a less common cause, which results in serious complications, such as intestinal necrosis. Thus, an accurate diagnosis of PSH confirmed on CT scans is important. We report a case of Richter's PSH in a patient who underwent RARP. The hernia was not confirmed on CT scans and was only diagnosed postoperatively.

## Case presentation

A 73‐year‐old man with an unremarkable medical history underwent RARP for prostate cancer. He had a body mass index of 22.2 kg/m^2^ and initial serum prostate‐specific antigen level of 4.3 ng/mL. Prostate biopsy revealed an adenocarcinoma, Gleason score 3 + 4 in three out of 10 sites. Metastasis was not detected on CT scans and bone scintigraphy. The patient was diagnosed with cT2aN0M0 prostate cancer.

**Fig. 1:**
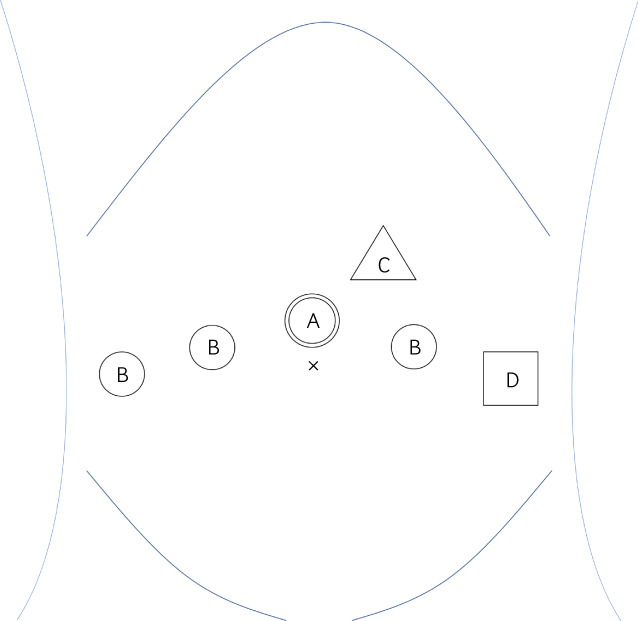
Trocar replacement for RARP. A: 12‐mm camera port. B: 8‐mm robot arm port. C: 5‐mm assistant port. D: 12‐mm assistant port.

RARP was performed via the trans‐peritoneal approach. The surgical trocars were placed as shown in Figure 1. The prostate was removed through the extended camera port. The fascia and peritoneum of the camera port were closed together with five interrupted sutures using 2–0 absorbable threads. The operation time and console time were 352 and 276 min, respectively. The blood loss was 300 mL.

**Fig. 2:**
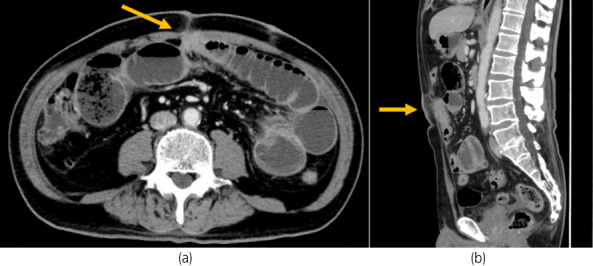
(a, b) Abdominal CT scans showed dilatation on the mouth side of the small intestine in contact with the camera port.

**Fig. 3:**
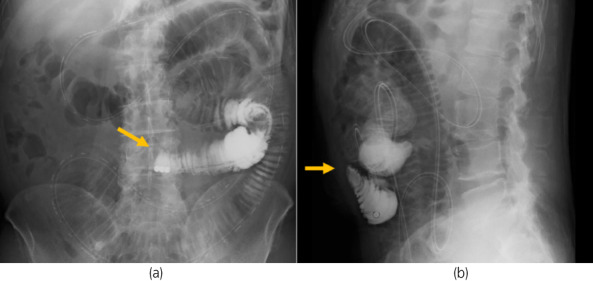
(a, b) Water‐soluble contrast from the ileus tube showed obstruction of passage in the small intestine adhering to the camera port. The tip of the tube is directly below the origin of the obstruction.

The patient had an uneventful postoperative course and was discharged 9 days after RARP. However, 11 days postoperatively, he reported abdominal pain, nausea, and vomiting. The CT scan revealed dilated small bowel loops proximal to the camera port (Fig. 2). An ileus tube was inserted, and water‐soluble contrast was injected into the tube. Obstruction just below the camera port was noted (Fig. 3). At this time, the patient was diagnosed with an adhesive ileus and was treated conservatively.

Ten days after ileus tube insertion (postoperative day 21), the patient's abdominal pain and nausea were relieved. The ileus tube was removed; however, the patient noted recurrence of nausea and vomiting. A plain abdominal radiograph showed dilated bowel loops.

**Fig. 4:**
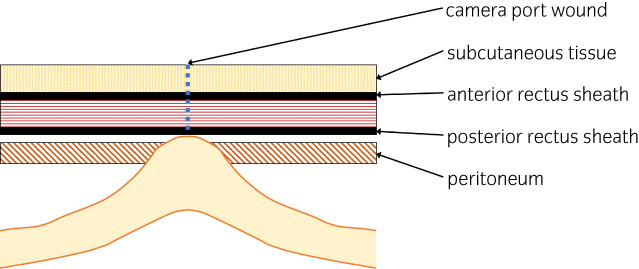
Schematic illustration of the hernia. Under the repaired fascia, a portion of the small intestine was incarcerated in the peritoneal defect.

Twenty‐two days postoperatively, laparotomy was performed considering the possible need for bowel resection due to adhesion. The wound of the camera port was reopened, and a portion of the small intestine was found incarcerated into the peritoneal defect despite repaired fascia (Fig. 4). These findings were consistent with Richter's hernia. The small bowel firmly adhered to the abdominal wall. The incarcerated portion of the small bowel was resected, and anastomosis was performed. The patient had a favorable recovery postoperatively.

## Discussion

Early postoperative small bowel obstruction occurs in 1–12% of abdominal operations, including laparoscopic and open surgeries.^1^ Its major causes include adhesions, internal hernias, and inflammation. Special attention should be paid to PSH after laparoscopic surgery.^2^ The prevalence of PSH following laparoscopic surgery is estimated to be approximately 0.5%,^3^ and that following RARP is reported to be 0.4–0.7%.^4^, ^5^ Tonouchi *et al*. reported that Richter's hernias frequently occur in the early postoperative period.^6^

Richter's hernia is a herniation of the anti‐mesenteric portion of the intestine. This condition does not always lead to complete intestinal obstruction. Thus, patients often complain of subclinical symptoms.^7^ In the present case, the patient temporarily recovered with conservative treatment, but his condition worsened after the removal of the ileus tube, suggesting a partial small bowel obstruction.

CT scans are the most commonly used diagnostic modality for PSH. However, it was ineffective in establishing the diagnosis of PSH in the present case. Several patients have been postoperatively diagnosed with Richter's hernia.^7^ Since Richter's hernia only involves a portion of the bowel wall, CT scans may yield a false‐negative result. In some cases, CT scans were unremarkable, and the diagnosis was confirmed only during the surgery. For example, obturator hernia is most often Richter's type. These lesions are not always diagnosed on CT scans.^8^

Water‐soluble contrast is used to differentiate partial small bowel obstruction from complete obstruction and to hasten the resolution of adhesive obstruction.^9^, ^10^ In this case, it was also useful in identifying the site of obstruction, and the minimal flow at the site of obstruction helped determine the cause.

In cases where the strangulation is not apparent, conservative treatment may be provided; however, surgery should still be considered. In early postoperative small bowel obstruction, the rate of improvement without surgery is reported to be 73–96% within 2 weeks.^11^, ^12^ Spontaneous lightening was reportedly unlikely beyond 10 days postoperatively.^11^ Since Richter's hernia is not always apparent on CT scans, surgery should always be considered in cases where conservative treatment is ineffective.

## Conclusion

We experienced a case of port site Richter's hernia in a patient who underwent RARP. Richter's hernia is difficult to diagnose on CT, and surgical intervention is necessary to treat the disease. This type of PSH should be considered in patients presenting with small bowel obstruction after laparoscopic surgery.

## Author contributions

Taisuke Tobe: Conceptualization; writing – original draft. Keiji Yuen: Writing – review and editing. Tomihiko Yasufuku: Supervision. Riku Uematsu: Supervision. Akira Miyazaki: Supervision. Masuo Yamashita: Supervision.

## Conflict of interest

The authors declare no conflict of interest.

## Approval of the research protocol by an Institutional Reviewer Board

N/A.

## Informed consent

Informed consent was obtained from the patients for publication of this case report.

## Registry and the Registration No. of the study/trial

N/A.
